# TRAF3: A novel regulator of mitochondrial physiology and metabolic pathways in B lymphocytes

**DOI:** 10.3389/fonc.2023.1081253

**Published:** 2023-01-27

**Authors:** Jaeyong Jung, Samantha Gokhale, Ping Xie

**Affiliations:** ^1^ Department of Cell Biology and Neuroscience, Rutgers University, Piscataway, NJ, United States; ^2^ Graduate Program in Cellular and Molecular Pharmacology, Rutgers University, Piscataway, NJ, United States; ^3^ Rutgers Cancer Institute of New Jersey, New Brunswick, NJ, United States

**Keywords:** TRAF3, mitochondria, metabolism, B lymphocytes, lymphomas

## Abstract

Mitochondria, the organelle critical for cell survival and metabolism, are exploited by cancer cells and provide an important therapeutic target in cancers. Mitochondria dynamically undergo fission and fusion to maintain their diverse functions. Proteins controlling mitochondrial fission and fusion have been recognized as essential regulators of mitochondrial functions, mitochondrial quality control, and cell survival. In a recent proteomic study, we identified the key mitochondrial fission factor, MFF, as a new interacting protein of TRAF3, a known tumor suppressor of multiple myeloma and other B cell malignancies. This interaction recruits the majority of cytoplasmic TRAF3 to mitochondria, allowing TRAF3 to regulate mitochondrial morphology, mitochondrial functions, and mitochondria-dependent apoptosis in resting B lymphocytes. Interestingly, recent transcriptomic, metabolic and lipidomic studies have revealed that TRAF3 also vitally regulates multiple metabolic pathways in B cells, including phospholipid metabolism, glucose metabolism, and ribonucleotide metabolism. Thus, TRAF3 emerges as a novel regulator of mitochondrial physiology and metabolic pathways in B lymphocytes and B cell malignancies. Here we review current knowledge in this area and discuss relevant clinical implications.

## Introduction

Tumor necrosis factor receptor-associated factor 3 (TRAF3), a cytoplasmic adaptor protein of the TRAF family, regulates the signal transduction pathways of a wide variety of immune receptors, including the TNF-R superfamily, lymphocyte antigen receptors, pattern recognition receptors (PRRs), and cytokine receptors (1–4). Through its scaffolding function and E3 ubiquitin ligase activity, TRAF3 differentially modulates a plethora of downstream signal transduction cascades, such as the activation of nuclear factor-κBs (NF-κB1 and NF-κB2), mitogen-activated protein kinases (MAPKs), and interferon-regulatory factors (IRFs), among others (1–4). Such regulatory function of TRAF3 exhibits an interesting dependence on the specific receptor engaged as well as the cellular context (1–4).

TRAF3 is ubiquitously expressed in various immune and non-immune cell types of mammals (1, 2, 5). Mice made genetically deficient in *Traf3* exhibit global defects and die by 10 days after birth (6). Subsequent studies of conditional *Traf3* knockout and cell type-specific *Traf3* transgenic mice revealed that TRAF3 plays critical and diverse roles in adaptive immunity and innate immunity as well as the homeostasis and stress responses of many tissues (1–4). Consequently, aberrant function of TRAF3 leads to a broad array of serious diseases in mouse models, including cancers, autoimmune diseases, inflammatory diseases, and infectious diseases (1–4, 7, 8).

Reinforcing the evidence obtained from mouse models, somatic mutations (such as homozygous deletions and inactivating mutations) of the *TRAF3* gene in humans were first identified in multiple myeloma (MM) and then other B cell malignancies (1, 3, 9–11). Somatic alterations of the *TRAF3* gene are also present in other human cancers (3). The first germline mutation of *TRAF3*, an autosomal loss-of-expression mutation (R338W), was initially reported in a patient with a history of herpes simplex virus-1 (HSV-1) encephalitis (12) and also detected in another patient with recurrent *Mycobacterium abscessus* infection (13). Interestingly, heterozygous germline mutations in *TRAF3* (premature stop codon mutations) were recently identified in 9 patients from five unrelated families, causing an immune dysregulation syndrome characterized by recurrent bacterial infection, autoimmunity, systemic inflammation, B cell lymphoproliferation, and hypergammaglobulinemia (14). Furthermore, genome-wide association studies (GWAS) and targeted analyses demonstrated that common genetic variants of *TRAF3*, which reduce TRAF3 expression, are associated with an increased risk of B cell malignancies, systemic lupus erythematosus, hypergammaglobulinemia, and recurrent bacterial infection in a wider population (14). Taken together, the above evidence highlights the importance of TRAF3 in the immune system, and particularly in B lymphocytes.

## TRAF3 in B cell biology and B cell malignancies

In normal B lymphocytes, TRAF3 is a critical regulator of mature B cell survival, B cell activation, and plasma cell differentiation and maturation (15–23). These important physiological functions of TRAF3 in B lymphocytes are achieved through its negative regulatory roles in the signal transduction pathways of multiple receptors that are central to B cell biology, including B cell antigen receptor (BCR), the co-stimulatory receptor CD40, receptors of the principle B cell survival factor BAFF, Toll-like receptors (TLRs), and IL-6 receptor (15–23). Specific deletion of the *Traf3* gene from B lymphocytes in mice leads to prolonged B cell survival, constitutive NF-κB2 activation, augmented BCR signaling, elevated T-dependent and T-independent antibody responses, enhanced TLR responses, and increased IL-6-induced plasma cell differentiation, which culminate in autoimmunity and B lymphomagenesis (15–24). Interestingly, transgenic overexpression of TRAF3 in B cells also renders B cells exhibiting enhanced reactions (also termed hyperreaction) to antigens and TLRs, resulting in autoimmunity and chronic inflammation (25). Thus, an appropriate level of TRAF3 proteins is required for normal B cell survival and functionality.

Consistent with the importance of TRAF3 in B cell biology, deletions and inactivating mutations of the *TRAF3* gene were first reported in human multiple myeloma (MM) (9, 10), a malignancy derived from plasma cells. According to the study by Keats et al., the deletion frequency of *TRAF3* is 15.8% in 158 analyzed MM patients (9). The high frequency of *TRAF3* deletions and inactivating mutations was verified in a larger cohort of patient study by Walker et al., which reported 16.7% genetic alterations of *TRAF3* in 463 examined MM patients, including 13% of deep deletions, 3.26% of mutations, and 0.43% of truncations of the *TRAF3* gene (26). Beyond genetic alterations, the Epstein-Barr virus (EBV)-encoded oncoprotein latent membrane protein 1 (LMP1) sequesters TRAF3 in B lymphocytes and can render EBV-infected wild type B cells functionally TRAF3-deficient, which may also contribute to the pathological mechanisms of B cell oncogenesis (27–29).

Subsequent studies revealed that deletions and inactivating mutations of *TRAF3* are frequently detected in many other types of mature B cell malignancies, including diffuse large B-cell lymphoma (DLBCL), splenic marginal zone lymphoma (MZL), B-cell chronic lymphocytic leukemia (B-CLL), mantle cell lymphoma (MCL), Waldenström’s macroglobulinemia, and Hodgkin lymphoma (HL) (1, 3, 9–11, 30). This is corroborated by frequent *TRAF3* mutations detected in canine non-Hodgkin lymphomas (NHL) (31–34). Similar to *Traf3* ^-/-^ mouse B cells, human B cells with germline *TRAF3* mutations that reduce TRAF3 expression also exhibit constitutive NF-κB2 activation and enhanced responses to BCR, BAFF, TLR9, and IL-6 signaling, including increased proliferation and plasma cell formation associated with elevated activation of NF-κB1, ERK, AKT, and STAT3 (14). Reconstitution of TRAF3 expression in TRAF3-deficient human MM cells induces apoptosis, demonstrating a tumor suppressive role of TRAF3 in B cell malignancies (9, 35). Of clinical significance, it is being appreciated that *TRAF3* alterations contribute to patient resistance to various therapies of B cell malignancies, including BTK inhibitors, PI3K inhibitors, proteasome inhibitors, HDAC inhibitors, cIAP antagonists, immunotherapy (*e*.*g*., rituximab), and chemotherapy (*e*.*g*., R-CHOP) (9, 19, 20, 36–39). In this context, a deeper understanding of the molecular mechanisms underlying TRAF3-mediated regulation of B lymphocytes is required to inform better treatment strategies for patients with B cell malignancies involving *TRAF3* deletions and other relevant alterations.

## TRAF3 is a novel regulator of mitochondrial physiology in B cells

While analyzing the subcellular distribution of TRAF3 in resting B lymphocytes, Liu et al. found that the majority of cytoplasmic TRAF3 is localized at the mitochondria in the absence of stimulation (40). They also noticed that BAFF-induced recruitment and subsequent degradation of TRAF3 mainly affect the proteins localized at the mitochondria in B cells (40). Given the central role of mitochondria in regulating apoptosis (41–43), Liu et al. pursued how TRAF3 is localized at mitochondria and what it does there.

Since TRAF3 does not contain any mitochondrial targeting motif or transmembrane domain, Liu et al. tested if TRAF3 interacts with mitochondrial outer membrane (MOM) proteins by employing a proteomic approach, including biochemical fractionation to isolate mitochondria and affinity purification to pull down mitochondrial TRAF3-interacting proteins followed by liquid chromatography-tandem mass spectrometry (LC-MS/MS)-based sequencing (40). To facilitate affinity purification, they transduced TRAF3-deficient human MM cells with lentiviruses expressing tagged TRAF3 (40). Liu et al. identified the MOM protein MFF (44) as a TRAF3-interacting protein and further verified the TRAF3-MFF interaction by co-immunoprecipitation and GST pull-down assays (40). MFF contains a coiled-coil domain that is known to mediate the interactions of other proteins with TRAFs (45–47). The domain structural analyses revealed that the TRAF-C domain of TRAF3 is required for binding to MFF, which was verified by specific pull-down of *in vitro* translated MFF by GST-TRAF3 but not by GST-TRAF3ΔTRAF-C (40). Furthermore, TRAF3 inhibits the phosphorylation and ubiquitination of MFF in resting B cells and co-transfected HEK293T cells, whereas overexpression of MFF leads to decreased ubiquitination of TRAF3 (40). Thus, MFF is a novel TRAF3-interacting protein that recruits TRAF3 to the MOM in B cells in the absence of receptor engagement.

The principal function of MFF is to promote mitochondrial fission, thereby contributing to the regulation of mitochondrial number, morphology, function, and quality (44, 48, 49). Consistent with the detected TRAF3-MFF interaction, increased protein levels of mitochondrial TRAF3 are associated with decreased mitochondrial number, altered mitochondrial morphology, reduced mitochondrial respiration, and increased mitochondrial ROS production and membrane permeabilization, which lead to caspase 9-dependent apoptosis in resting wild type B cells (40). Liu et al. and Mambetsariev et al. found that deletion of TRAF3 has the opposite effects on mitochondrial morphology, respiration, ROS production, and mitochondria-dependent apoptosis in resting B cells (40, 50). Interestingly, lentivirus-mediated overexpression of MFF restores mitochondria-dependent apoptosis in TRAF3-deficient human MM cells (40). Corroborating these findings, Rae et al. recently reported that B lymphoblastoid cell lines (BLCLs) derived from patients with germline premature stop codon mutations of *TRAF3* display an increased oxygen consumption rate, indicative of elevated mitochondrial respiration, which is accompanied by altered mitochondrial morphology, up-regulated COX II expression and enhanced cytochrome c oxidase activity (14). Reconstitution of TRAF3 expression in patient-derived BLCLs inhibits mitochondrial respiration and the expression of the mitochondrial co-transcriptional regulator PGC1α (14). Moreover, transgenic overexpression of *TRAF3* in B cells also promotes NHL development in mice when the anti-apoptotic protein BCL-2 is simultaneously overexpressed (51), suggesting a need for BCL-2-mediated protection of mitochondria in TRAF3-overexpressing B cells. Therefore, TRAF3 is a novel regulator of mitochondrial physiology in normal and malignant B cells.

It is noteworthy that BAFF inhibits mitochondrial ROS production and prevents mitochondria-dependent apoptosis in wild type (WT) but not *Traf3* ^-/-^ B cells (40). Upon BAFF stimulation, BAFF-Rs recruit TRAF3 from the MOM to plasma membrane, which would lead to disruption of the TRAF3-MFF interaction and therefore modulate mitochondrial functions in WT B cells (40). Interestingly, the TRAF3-MFF interaction appears to result in decreased phosphorylation and ubiquitination of MFF as well as decreased ubiquitination of TRAF3 (40). Phosphorylation of MFF has been shown to increase the activity of MFF in recruiting Drp1, the GTPase that executes mitochondrial fission, to the MOM, promoting mitochondrial fission (52–55). Ubiquitination of MFF may enhance clearance of damaged mitochondria *via* mitophagy or may induce the degradation of MFF under non-stressed conditions (56, 57). Detailed mechanisms of how TRAF3 inhibits the phosphorylation and ubiquitination of MFF in resting B cells remain unclear. TRAF3 is known as an E3 ubiquitin ligase. Other E3 ubiquitin ligases that can interact with MFF include Parkin and MARCH5 (56–58). However, only Parkin has been shown to directly catalyze the ubiquitination of MFF (56, 57). Deubiquitinating enzymes of MFF have not been reported yet. It is possible that TRAF3 may catalyze the ubiquitination of Parkin or relevant deubiquitinating enzymes, kinases or phosphatases to indirectly inhibit the ubiquitination and phosphorylation of MFF in resting B cells. Alternatively, the TRAF3-MFF interaction may interfere with the accessibility of MFF by Parkin and kinases or facilitate the recruitment of relevant deubiquitinating enzymes or phosphatases, leading to reduced ubiquitination and phosphorylation of MFF. Such detailed mechanisms await further investigation.

An interesting open question is whether TRAF2, another member of the TRAF family that has overlapping functions with TRAF3 in B cells, can also interact with MFF. The TRAF3-TRAF2 heterotypic interaction is known to bridge the formation of the cIAP1/2-TRAF2-TRAF3-NIK complex in B cells (1). The TRAF3-TRAF2 interaction minimally involves the TRAF-C domain of TRAF3 and the TRAF-N domain and zinc fingers 4 and 5 of TRAF2 (59). It remains unknown whether the TRAF3-MFF interaction interferes with the TRAF3-TRAF2 interaction when TRAF3 proteins are limiting in B cells. Following viral infection, both TRAF3 and TRAF2 are recruited to the mitochondrial antiviral signaling complexes by MAVS (60–63), while in response to ER stress, TRAF2 is translocated to ER *via* interacting with the ER stress sensor IRE1α (64, 65). In addition, cIAP1/2 also binds to caspases and upon mitochondrial membrane permeabilization, cIAP1/2 binds to Smac that is released from mitochondrial intermembrane space to cytosol (66, 67). However, it is unclear whether and how the TRAF3-MFF, TRAF3-TRAF2, and TRAF2-cIAP1/2 interactions are affected by viral infection, ER stress, and mitochondrial membrane permeabilization in B cells. These unanswered questions await further investigation.

## TRAF3 regulates specific metabolic pathways in resting B cells

The metabolic mechanisms underlying TRAF3-mediated regulation of B cells have just begun to be unraveled. To understand the metabolic basis of TRAF3-mediated regulation of B cell survival, we recently exploited multiple “omics” approaches, including metabolomics, lipidomics, and transcriptomics (35). Integrated analyses of these “omics” datasets revealed that TRAF3 regulates specific metabolic pathways in resting B cells, including phospholipid, glucose, and ribonucleotide metabolism (35).

**Figure 1:**
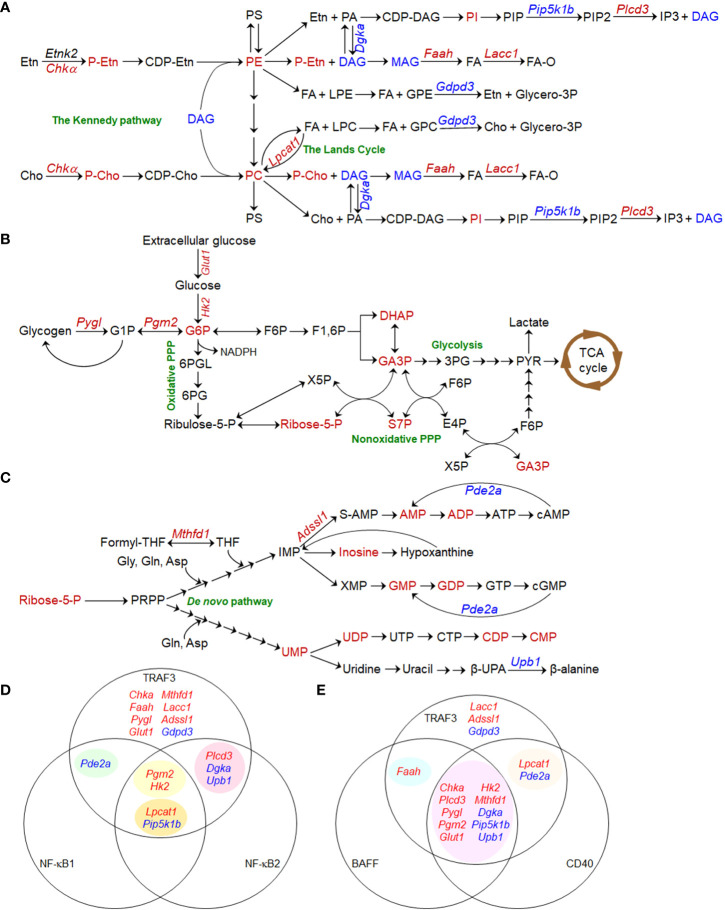
Metabolic pathways regulated by TRAF3 in B lymphocytes. **(A–C)** Pathway schematics showing TRAF3-mediated metabolic regulation in B cells. Small metabolites, lipids, transporters, and metabolic enzymes that are dysregulated in *Traf3* ^-/-^ B cells are shown in red (for up-regulated) or blue (for down-regulated). Enzymes and transporters are denoted in *Italic* font in the schematics. **(A)** The interconnected phosphatidylcholine (PC) and phosphatidylethanolamine (PE) metabolic pathways. **(B)** Glucose metabolic pathways. **(C)** Ribonucleotide metabolic pathways. **(D, E)** Venn diagram of TRAF3-regulated metabolic genes and those regulated by NF-κB1 and NF-κB2 **(D)** or BAFF and CD40 **(E)**. Metabolic genes regulated by NF-κB1, NF-κB2, BAFF or CD40 are extracted from the published Gene Expression Omnibus (GEO) datasets GSE75761, GSE75762, GSE58972, and GSE62559. **(D)** TRAF3 regulates the expression of 8 metabolic genes *via* NF-κB-independent mechanisms. NF-κB2 and NF-κB1 act synergistically to promote *Lpcat1* expression and suppress *Pip5k1b* expression. In contrast, *Pgm2* and *Hk2* expression are only inhibited by compound deletion of both *Relb* and *cRel*, suggesting redundant roles of NF-κB2 and NF-κB1 in up-regulating the expression of these two enzymes. **(E)** The majority (10/16) of the differentially expressed metabolic genes identified in *Traf3* ^-/-^ B cells are consistently changed in WT B cells following BAFF and CD40 stimulation. TRAF3 also regulates the expression of 3 unique enzymes, which are not affected by BAFF or CD40 stimulation.

We found that a variety of metabolites, lipids and enzymes regulated by TRAF3 in B cells are clustered in the interconnected phosphatidylcholine (PC) and phosphatidylethanolamine (PE) metabolic pathways (**Figure 1A**) (35). Enzymes that are regulated by TRAF3 and may contribute to the altered PC and PE metabolism include choline kinase α (Chkα), lysophosphatdiylcholine acyltransferease 1 (Lpcat1), glycerophosphodiester phosphodiesterase3 (Gdpd3), diacylglycerol kinase α (Dgkα), and fatty acid amide hydrolase (Faah), etc. (35, 68). Using stable isotope labeling, we demonstrated that Chkα-driven *de novo* biosynthesis of PC is remarkably elevated in *Traf3* ^-/-^ mouse B cells and decreased in TRAF3-reconstituted human MM cells containing biallelic *TRAF3* deletions (35). Inhibition of Chkα by RSM932A (also named TCD-717) or MN58B substantially reverses the survival phenotype of TRAF3-deficient B cells both *in vitro* and *in vivo* (35). Thus, TRAF3-regulated choline metabolism has diagnostic and therapeutic value for B cell malignancies with *TRAF3* deletions or relevant alterations (35, 68).

We observed significant elevation of 5 glucose metabolic intermediates in resting *Traf3* ^-/-^ B cells, including glucose-6-phosphate (G6P), the convergence point of the glycolytic and pentose phosphate pathways (PPP) (69), and 4 metabolites of nonoxidative PPP (35). Our transcriptomic analysis identified up-regulation of two key enzymes in *Traf3* ^-/-^ B cells: phosphoglucomutase 2 (Pgm2) and glycogen phosphorylase L (Pygl) that is responsible for glycogen breakdown (70, 71). Mambetsariev et al. reported that glucose transporter 1 (Glut1) and hexokinase II (HKII) are also up-regulated in *Traf3* ^-/-^ B cells and that these cells exhibit increased glucose uptake (50). Inhibition of glucose metabolism by the Glut1 inhibitor STF-31 or the glycolysis inhibitor 2-deoxyglucose (2-DG) suppresses B cell survival, while glucose supplementation in serum-free medium is required for long-term survival of *Traf3* ^-/-^ B cells in culture (50). These findings are strengthened by the evidence that B cell-specific deletion of Glut1 leads to substantially decreased numbers of peripheral B cells in mice (72) and that Glut1 expression is necessary to maintain elevated glucose metabolism and to promote cell survival of human MM and B cell acute lymphoblastic leukemia (B-ALL) (73, 74). Therefore, TRAF3 can regulate both glycogen breakdown and glucose uptake to modulate glucose metabolism (**Figure 1B**), which also affects B cell survival.

In line with elevation of ribose-5-phosphate (Ribose-5-P), a metabolite generated by nonoxidative PPP that serves as the molecular backbone of ribonucleotide biosynthesis (69, 75–77), we detected significantly increased levels of 9 ribonucleotides in resting *Traf3* ^-/-^ B cells (**Figure 1C**) (35). We also identified up-regulation of two enzymes responsible for ribonucleotide biosynthesis (Mthfd1 and Adssl1) as well as down-regulation of two enzymes involved in ribonucleotide catabolism (Upb1 and Pde2a) in *Traf3* ^-/-^ B cells (35). However, we did not observe significant changes in ribonucleotide triphosphates (35), probably because they are consumed to support elevated transcription and other reactions required for the prolonged B cell survival. MTHFD1, an enzyme crucial for *de novo* purine biosynthesis, is up-regulated in human MM, NHL, and HL (Oncomine) (78–81). Interestingly, a common polymorphism of MTHFD1 R653Q (*MTHFD1* G1958A) in the synthetase domain impairs purine synthesis and the corresponding AA genotype is associated with a decreased risk of human B-ALL and NHL (82, 83), indicating a role of this enzyme in B cell oncogenesis. Taken together, elevated PC and PE synthesis, glucose metabolism, and ribonucleotide synthesis are the metabolic basis mediating the aberrant survival of TRAF3-deficient B cells.

BAFF-R or CD40 signaling recruits TRAF3 to receptor complexes at plasma membrane rafts, inducing TRAF3 degradation, NF-κB2 activation, and B cell survival (1). TRAF3 also inhibits NF-κB1 activation induced by CD40 and BCR signaling (18, 19). We thus analyzed the Gene Expression Omnibus (GEO) datasets for relevant metabolic genes in mouse B cells genetically deficient in different subunits of NF-κB in comparison to WT B cells, including *Nfkb2* ^-/-^ (GSE75761 and GSE75762), *Rela* ^-/-^ (GSE58972), *cRel* ^-/-^, and *Relb* ^-/-^ *cRel* ^-/-^ (GSE62559) B cells, in the absence or presence of stimulation with BAFF, CD40 or CD40 plus IgM (84–86). The results of our analyses revealed that 8 of the 16 TRAF3-regulated metabolic genes (35, 50) are independent of NF-κBs (**Figure 1D**). Down-regulation of *Pde2a* is dependent on NF-κB1, while regulation of 3 other genes is dependent on NF-κB2. Interestingly, NF-κB2 and NF-κB1 act synergistically to promote *Lpcat1* expression and suppress *Pip5k1b* expression. In contrast, NF-κB2 and NF-κB1 appear to play redundant roles in up-regulating the expression of *Pgm2* and *Hk2*, which is only inhibited by compound deficiency in both *Relb* and *cRel* (84–86). On the other hand, the majority (10/16) of the metabolic genes differentially expressed in *Traf3* ^-/-^ B cells are consistently changed in WT B cells following BAFF and CD40 stimulation (**Figure 1E**) (84–86). *Faah* and 2 other regulated genes (*Lpcat1* and *Pde2a*) are only shared with either BAFF or CD40 stimulation, respectively. However, 3 additional genes (*Lacc1*, *Adssl1*, and *Gdpd3*) are uniquely altered by *Traf3* deficiency. Thus, although similar to BAFF- or CD40-induced physiological B cell survival, aberrant survival of TRAF3-deficient B cells exhibits certain distinct features in metabolic reprogramming.

## Discussion

**Figure 2:**
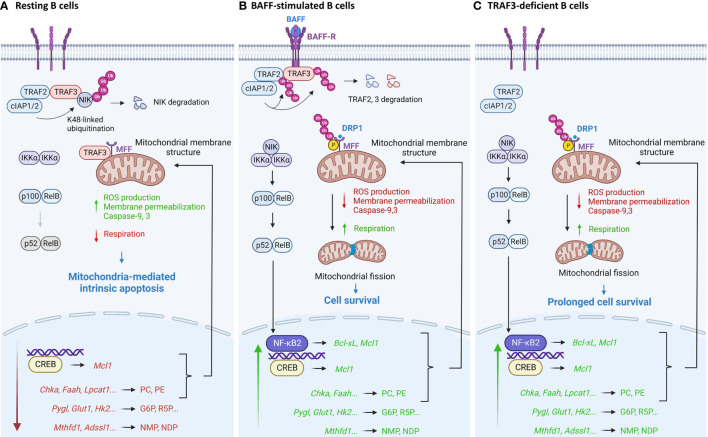
Pathway model depicting TRAF3-mediated regulation of mitochondria-dependent apoptosis in B lymphocytes. **(A)** In resting B cells, TRAF3 regulates mitochondrial physiology and metabolic pathways to promote mitochondria-dependent apoptosis. TRAF3 controls mitochondrial morphology and function *via* its direct interaction with MFF to inhibit MFF phosphorylation and ubiquitination, *via* metabolic reduction of phospholipids PC and PE to alter mitochondrial membrane structure, and *via* inhibition of the NIK-NF-κB2 and nuclear CREB pathways to down-regulate the expression of anti-apoptotic proteins such as Mcl1 and Bcl-xL. In addition, TRAF3-mediated repression of glucose and ribonucleotide metabolism also coordinately suppresses B cell survival. **(B)** BAFF stimulation recruits TRAF3 to membrane rafts and subsequently induces TRAF3 degradation, thereby reversing many TRAF3-mediated effects to promote B cell survival. Separation of TRAF3 from MFF at the MOM also results in increased phosphorylation of MFF, leading to enhanced activity of MFF in recruiting Drp1, the GTPase that executes mitochondrial fission, to the MOM, promoting mitochondrial fission. **(C)** Disruption of all the TRAF3-dependent mechanisms in TRAF3-deficient B cells leads to prolonged survival independent of BAFF, which eventually contributes to B cell malignant transformation. This figure was created with BioRender.com.

In summary, recent proteomic and metabolic evidence reveals that TRAF3 regulates mitochondrial physiology and metabolic pathways to control B cell survival (**Figure 2**). TRAF3 can regulate mitochondrial morphology and function *via* interacting with MFF (40). Interestingly, TRAF3-mediated metabolic regulation leads to reduced levels of the phospholipids PC and PE (35), which are the most abundant phospholipids of mitochondrial membranes – comprising ∼40% and ∼30% of total mitochondrial phospholipids, respectively (87). Moderate dysregulation of PC and PE has profound effects on mitochondrial physiology and mitochondria-dependent apoptosis (88, 89). Moreover, the expression of anti-apoptotic proteins such as Mcl1 and Bcl-xL, critical regulators of mitochondrial physiology and intrinsic apoptosis (90, 91), is inhibited by TRAF3 *via* the downstream NIK-NF-κB2 and nuclear CREB pathways (16, 17, 92, 93). Therefore, TRAF3 can regulate mitochondrial physiology and mitochondria-dependent apoptosis in B cells *via* multi-layered mechanisms. In addition, TRAF3 also regulates the expression of key enzymes responsible for glucose and ribonucleotide metabolism (35, 50), which coordinately provide the metabolic basis to control B cell survival.

The above proteomic and metabolic findings have therapeutic implications, suggesting that manipulation of mitochondrial dynamics and inhibition of key metabolic enzymes or transporters offer new perspectives of treatment strategies for B cell malignancies, especially those with *TRAF3* deletions or relevant alterations. Indeed, we demonstrated that overexpression of MFF restores the intrinsic apoptosis in TRAF3-deficient human MM cells, supporting a therapeutic potential of drugs that have been developed to target mitochondrial dynamics and are being tested in other disease models, including cell permeable peptidomimetics of MFF, mitochondrial division inhibitor-1 (mdivi-1), dynasore, P110, and 15-oxospiramilactone, etc. (44, 48, 94, 95). We also showed that inhibition of choline metabolism by the Chkα inhibitors TCD-717 or MN58B substantially reduces the expanded B cell compartment in B-*Traf3* ^-/-^ mice and induces apoptosis in TRAF3-deficient human MM cells (35), while Mambetsariev et al. demonstrated that inhibition of glucose metabolism by the Glut1 inhibitor STF-31 or the glycolysis inhibitor 2-DG dampens *Traf3* ^-/-^ B cell survival (50). Moreover, available information suggests that several other TRAF3-regulated metabolic enzymes (*e*.*g*., Lpcat1, Pygl, Hk2, and Mthfd1) are also targetable points in cancers (70, 82, 83, 96–104). Thus, all these mitochondria-targeting drugs and pharmacological inhibitors of metabolic enzymes/transporters can be exploited, alone or in combination with current therapies, for the treatment of human B cell malignancies to improve patient outcome.

Increasing evidence indicates that TRAF3 is a tumor suppressor not only in B cell malignancies, but also in a variety of cancers derived from macrophages, osteoblasts, and epithelial cells of different tissues. Examples include histiocytic sarcoma, osteosarcoma, head and neck cancer, bladder cancer, colorectal cancer, breast cancer, liver cancer, and lung cancer (3, 7, 8, 105–112). Loss of TRAF3 also leads to constitutive NF-κB2 activation in macrophages, osteoblasts/osteoclasts, and epithelial cells (7, 107, 111–114). Similar to that observed in B cells, TRAF3 also regulates cell survival and mitochondrial ROS production in macrophages and epithelial carcinoma cells under specific circumstances (109, 115). We detected co-immunoprecipitation of TRAF3 with MFF in transfected HEK293T epithelial cells. Interestingly, Liu et al. recently reported that TRAF3 interacts with the mitochondrial fusion protein mitofusin-1 (MFN1) in ovarian cancer cells, which is enhanced upon TLR4 signaling induced by Selene nanoparticles (116). Furthermore, Zhou et al. previously reported co-immunoprecipitation of TRAF3 with the MOM protein PINK1 in resting primary mouse peritoneal macrophages (117). Therefore, whether and how TRAF3 regulates mitochondria-dependent apoptosis in these cell types ***via*** the TRAF3-MFF axis and metabolic mechanisms as described for B cells or ***via*** distinct MFN1- and PINK1- dependent mechanisms are significant areas for future exploration. Such knowledge would lay the foundation for testing the therapeutic potential of *TRAF3* gene therapy, mitochondria-targeting drugs, and pharmacological inhibitors of metabolic enzymes/transporters in histiocytic sarcoma, osteosarcoma, and epithelial cell-derived cancers of various tissues, especially those with *TRAF3* deletions or relevant alterations.

## Author contributions

All authors listed have made a substantial, direct, and intellectual contribution to the work and approved it for publication.
